# Peer Review of “Real-World Performance of COVID-19 Antigen Tests: Predictive Modeling and Laboratory-Based Validation”

**DOI:** 10.2196/83479

**Published:** 2025-10-06

**Authors:** Gerald Kost

**Affiliations:** 1 UC Davis, Davis, CA, United States

**Keywords:** COVID-19, real-world data, limit of detection lateral flow test, probability of positive agreement, logistic regression, COVID-19 antigen test clinical performance

*This is a peer-review report for “Real-World Performance of COVID-19 Antigen Tests: Predictive Modeling and Laboratory-Based Validation*.*”*

## Round 1 Review

### General Comments

This paper [1] introduces a novel approach to speeding COVID-19 antigen test deployment, possibly during the next pandemic, whether it be a COVID-19 variant or a new pathogen that arises.

### Specific Comments

#### Major Comments

1. The authors’ clinical conclusions based on their prediction theory are overly optimistic.

2. The authors can explore actual clinical evaluations to determine the robustness of their prediction modeling.

3. Thus, the paper merits publication, providing the limitations are more clearly described and the conclusions are limited to the mathematical results for which the authors have proven their claims theoretically. Extension to clinical applicability is a different story yet to be told.

4. The authors should be encouraged to move forward in view of the need and the poor performance of COVID-19 rapid antigen tests during the pandemic because of low sensitivity, a lack of deep understanding, and the “prevalence boundary,” a measure of when the rate of false omissions becomes too high and false negatives spread disease.

#### Minor Comments

5. Needs English grammar review. This could be achieved by using an artificial intelligence editor.
